# A Symmetric Image Encryption Algorithm Based on a Coupled Logistic–Bernoulli Map and Cellular Automata Diffusion Strategy

**DOI:** 10.3390/e21050504

**Published:** 2019-05-17

**Authors:** Wei Zhang, Zhiliang Zhu, Hai Yu

**Affiliations:** Software College, Northeastern University, Shenyang 110819, China

**Keywords:** image privacy, evolution effects in cellular automata, chaotic system, logistic–Bernoulli map

## Abstract

In this paper, the properties of the classical confusion–substitution structure and some recently proposed pseudorandom number generators using one-dimensional chaotic maps are investigated. To solve the low security problem of the original structure, a new bit-level cellular automata strategy is used to improve the sensitivity to the cryptosystem. We find that the new evolution effects among different generations of cells in cellular automata can significantly improve the diffusion effect. After this, a new one-dimensional chaotic map is proposed, which is constructed by coupling the logistic map and the Bernoulli map (LBM). The new map exhibits a much better random behavior and is more efficient than comparable ones. Due to the favorable properties of the new map and cellular automata algorithm, we propose a new image-encryption algorithm in which three-dimensional bit-level permutation with LBM is employed in the confusion phase. Simulations are carried out, and the results demonstrate the superior security and high efficiency of the proposed scheme.

## 1. Introduction

Information security plays a significant role in peoples’ digital lives. With the development of network technologies and wide use of portable digital devices, information is increasingly transmitted or shared via the Internet. Meanwhile, theories and protocols have been established and applied to guarantee the security of distributed information. In recent years, increasing amounts of multimedia information are shared via the Internet, including photos, voice data, and short videos, which have different characteristic features to text information. Such information is easily understood and vividly representative. Unlike text-based information, multimedia information is always highly redundant in the time and space domains, and traditional ciphers, such as DES (Data Encryption Standard) and AES (Advanced Encryption Standard), are inefficient in dealing with multimedia information to satisfy the real-time requirements of multimedia information transmission.

To cope with this issue, researchers have proposed many ciphers based on different perspectives, which provide high security and efficiency for multimedia information. For instance, DNA coding-based ciphers [1,2], S-box-based ciphers [3], compressive sensing-based ciphers [4,5], wave function-based ciphers [6], etc. Moreover, by virtue of the similitude to pixel matrix of digital image, Sudoku matrix [7] and Latin square [8] have also been utilized in image ciphers. Originating in the late 1990s, chaos theory-based encryption algorithms have developed significantly, and most of them employ a confusion–diffusion structure, which was first proposed by Fridrich [9]. Due to their superior properties, such as ergodicity, sensitivity to initial conditions and parameters, and dynamical complexity, chaotic systems are quite similar to cryptosystems, and they are good candidates for generating pseudorandom numbers when applying confusion and diffusion operations. 

In recent years, many relevant works have been reported, and they can be mainly divided into four categories. 

In the first category, the properties of chaotic systems are investigated. Their benefits and drawbacks are analyzed, and comparisons among different chaotic systems are studied. In addition, new chaotic maps are proposed. In [10], Zhou et al. proposed a new one-dimensional chaotic map by combining two existing one-dimensional chaotic maps as a pseudorandom number generator and facilitated a new image-encryption algorithm using one-dimensional substitution and image-rotation schemes. In [11], Hua et al. proposed a new scheme to formulate a new 2D chaotic map by combining two 1D chaotic maps, and after that, a new chaotic magic transform is used to facilitate the confusion operations. In [12], a new 2D chaotic logistic-adjusted-sine map was proposed, and its chaotic properties were deeply investigated. With the help of the new chaotic map, a new image-encryption scheme, LASIES, was proposed. The traditional 2D chaotic maps, such as the standard map or cat map, have some problems when applied in the confusion stage of a cryptosystem, such as the fixed-point problem, repeated-pattern problem, and short-cycle problem. In [13,14], the periodic distribution of the generalized discrete cat map was analyzed. In [15], a new pattern that makes nonlinear combination of two 1D chaotic maps was proposed, and a sine–logistic map (SLM) was attained by the pattern, then applied to the encryption process.

In the second category, the researchers focus on encryption algorithms in the space domain without transformations. Two branches can be found in this category: pixel-level ciphers and bit-level ciphers. Pixel-level image-encryption schemes are significantly researched. In this branch, the Fridrich architecture or its transformed structure is used, and in its confusion and diffusion stages, all operations are performed on the pixel level. In [16], two random sequences were firstly generated, and after that, the mapping rules were obtained by sorting the two random sequences and searching each number. The columns and rows are the basic units during the permutation stage, according to the mapping rules. In the diffusion phase, addition and modulation are applied to pixel values, random values, and previous-cipher pixel values. Both confusion and diffusion are performed on the pixel level. In [17], with the help of the reverse 2D chaotic map, a new mechanism to randomly visit the plain image was proposed. In the permutation stage, a new mapping rule was employed from a random position in the plain image to another random position in the cipher image, and the two random positions were generated by different 2D chaotic maps. In the diffusion phase, the logistic map governs the process. In [18], the permutation stage is realized by the vertical and horizontal wave-lines generated by Arnold map. At the diffusion stage, blocks of pixels were consecutively diffused, and the current diffused block will influence the next block. In the second branch of this category, the cryptosystem was implemented on the bit level. Due to its special properties, bit-level permutation has a better confusion effect than pixel-level permutation. In [19], bit-level permutation was firstly proposed. In the first step of bit-level permutation, the image was divided into N bit planes according to the bit length of each pixel in the plain image. For example, a 256-grey-level plain image has eight-bit planes. In the second step, the higher four-bit planes were permuted independently as they contain more than 94% of the total information, while the lower four-bit planes were permuted together since the contain only 4.4% of the total information. Since the bit constitution of each pixel is changed, their locations and values are encrypted. In [20], the different properties of different bit planes were investigated, and a new expand-and-shrink bit-level permutation algorithm was proposed. In [21], the plain image was addressed as a 3D bit matrix, and three orthogonal Latin cubes were utilized to achieve spatial permutation and diffusion in the 3D bit matrix.

In the third category, optics techniques are employed to facilitate the image-encryption algorithm. There are usually three steps in optics-based image-encryption algorithms. In the first step, the plain image is divided into a series of elemental images by light propagation using a lenslet or pinhole array. Each of the elemental images contains most of the plain-image information. In the second step, encryption operations are applied on these elemental images, and in the last step, these encrypted elemental images are back-propagated by the lenslet array or reconstructed by a computational integral-imaging reconstruction algorithm to obtain the final cipher text. In [22], Li et al. proposed a new optical-based image-encryption algorithm, in which cellular automata and a chaotic system were employed to generate random sequences. One sequence was used to encrypt the pixel values in the elemental images, while another sequence was sorted and searched to generate the mapping rule for permuting each elemental image. In [23], a new multiple-image simultaneous encryption algorithm using a back-propagation neural network (BP neural network) was proposed to solve the security and interference problems.

In the fourth category, all image-encryption operations are performed in the frequency domain or transformed domain. The plain image is firstly transformed into the frequency domain, using a method such as the DCT (Discrete Cosine Transform) transform, wavelet transform, or Fourier transform. In the frequency domain, the information of the plain image is not evenly distributed, and most of the information is concentrated in a finite number of pixels. Selective encryptions are usually applied on this finite number of pixels in terms of low-frequency pixels. In [24], a new lossless image-encryption algorithm was proposed. In this cipher, the image was firstly transformed using the two-dimensional Haar wavelet transform, and the permutation operations were performed in the frequency domain. In the second step, the inverse Haar wavelet transform was applied and diffusion operations were performed in the space domain.

The image-encryption algorithms that belong to the different categories provide different ways to enhance the security of multimedia information. Most algorithms in all categories employ a permutation and diffusion architecture. However, the permutation algorithms always suffer from repeated-pattern problems for permuted images [25], which indicates that random permutation is not sufficient. In the diffusion phase, most of the algorithms choose a one-dimensional chaotic map and apply exclusive-or operations on the transformed one-dimensional pixel array. Diffusion operations of this type are classical and fast. However, they do not provide sufficient sensitivity to slight modifications of the plain image. This sensitivity is crucial for protecting against differential attacks and can be measured by NPCR (number of pixels change rate) [25], which is the different pixels’ percentage in two images of the same size. In an ideal situation, a slight modification to the plain image can lead to different values of 99.6% of the pixels after one or two encryption rounds. In other words, the diffusion algorithm should spread one-bit modification to most of the remaining pixels in one diffusion round, and the classical diffusion algorithms, which are employed by most image-encryption algorithms, cannot achieve this goal, requiring more encryption rounds to diffuse the modification.

To cope with these problems, a new 3D bit-level permutation scheme was proposed to eliminate the repeated patterns in the permuted image. To further enhance the security of the random sequences used in the permutation and diffusion stages, a new chaotic map logistic–Bernoulli map (LBM) was proposed and investigated, which exhibits much better chaotic behavior than other one-dimensional chaotic maps. In the diffusion phase, a new bit-level cellular automata strategy was investigated to improve the sensitivity to the plain image modification. We found that the new evolution effects among different generations of the cells in cellular automata can significantly improve the diffusion effect of the cryptosystem. 

The rest of the paper is organized as follows. In Section 2, the new chaotic map and its properties are introduced. In Section 3, a cellular automata-based diffusion strategy is described. The proposed cryptosystem is described in Section 4. In Section 5, simulation results and the performance analysis are reported, and in the last section, a conclusion is drawn.

## 2. A New One-Dimensional Chaotic Map for Image Encryption

One-dimensional (1D) maps are usually used as pseudorandom number generators in image-encryption algorithms due to their simplicity and efficiency. The logistic map, sine map, tent map, and Bernoulli map [26] are four 1D maps that are commonly employed in cryptosystems, and their definitions are as follows.

(1) $$x_{n+1}=\delta \left(\mu ,x_{n}\right)=\mu x_{n}\left(1-x_{n}\right),$$

(2) $$x_{n+1}=\beta (a,x_{n})=a\sin(\pi x_{n})/4,$$

(3) $$x_{n+1}=\eta (u,x_{n})=\left\{ \begin{array}{l} ux_{n}/2,x_{i}<0.5\\ u(1-x_{n})/2,x_{i}\geq 0.5 \end{array}\right.,$$

(4) $$x_{n+1}=B(\sigma ,x_{n})=\sigma x_{n}\mathrm{mod}1=\left\{ \begin{array}{l} \sigma x_{n},0\leq x_{i}<0.5\\ \sigma x_{n}-1,0.5\leq x_{i}<1 \end{array}\right.,$$

However, single 1D maps suffer from the following problems [10]: (1) the chaotic behaviors are not continuous; (2) the attack on the random sequence generated by a 1D map is computationally inexpensive and fast; and (3) the output sequence is not uniformly distributed. As a consequence, many researchers focus on high-dimensional chaotic systems to facilitate image-encryption operations [24]. Although high-dimensional chaotic systems provide more complex structures and chaotic behaviors, their software or hardware implementations are complex and expensive [11], which indicates that a pseudorandom number generator that uses a high-dimensional chaotic map is inefficient. To achieve a balance between efficiency and security, in [10] and [11], Zhou and Hua suggested coupling or combining two or more 1D chaotic maps to form a new 1D or multi-dimensional chaotic system. On the one hand, a coupled high-dimensional chaotic map keeps the system computationally inexpensive and highly efficient; on the other hand, it has been demonstrated that a coupled system has better chaotic behavior and a larger Lyapunov exponent [10,11,12].

The speed of iterating the chaotic map to generate pseudorandom numbers is significant, since it constitutes a large amount of the encryption time. The logistic map, sine map, tent map, and Bernoulli map have similar chaotic behavior, especially as pseudorandom number generators in image-encryption operations. However, their speeds vary. To examine this property, we performed calculations with each chaotic map to generate 3,000,000 random numbers 10 times. The simulation environment was a personal computer with Windows 7 Ultimate, Intel 3.4 GHz Dual-core CPU, and 8 GB memory, and the compiling environment is Visual C++ 2010. The average data are listed in Table 1. 

As Table 1 illustrates, to generate 3,000,000 pseudorandom numbers (without quantification), the logistic map was much faster than the sine map, and a little faster than the tent map. Although the logistic map, sine map, and tent map were all very fast, the logistic map had the highest speed. This is due to its simple structure; moreover, to calculate each number, only multiplication and addition operations are required. The sine map was the slowest, due to the relatively complex trigonometric function calculation in each iteration. The tent map was relatively slow due to the condition determination for the piecewise function in each iteration.

In [10], Zhou et al. proposed a new scheme to combine two 1D chaotic maps as seed maps to form new 1D chaotic maps. The new maps were the logistic–tent system (LTS; the logistic map and tent map are the two seed maps), logistic–sine system (LSS; the logistic map and sine map are the two seed maps), and tent–sine system (TSS; the tent map and sine map are the two seed maps). Simulation results show that the new 1D chaotic maps, LTS, LSS, and TSS, have better chaotic behaviors and larger Lyapunov exponents.

As Table 1 shows, the sine map and tent map are relatively slow, and provide chaotic behaviors similar to that of the logistic map. To achieve a good balance between security and efficiency for image encryption, a new one-dimensional chaotic system joint logistic map and Bernoulli map was proposed. The logistic–Bernoulli map (LBM) is defined in Equation (5).

(5) $$x_{n+1}=f\left(x_{n},\gamma \right)=\{\begin{array}{c} \gamma \times x_{n}\times \left(1-x_{n}\right)+\left(8-\gamma \right)\times x_{n},x_{n}\leq 1\\ 1-\gamma \times x_{n}\times \left(1-x_{n}\right)-\left(8-\gamma \right)\times x_{n},x_{n}>1 \end{array},$$

where the parameter *y* ∈ (0, 4], and *x*_0_ is the initial value of the LBM.

**Figure 1:**
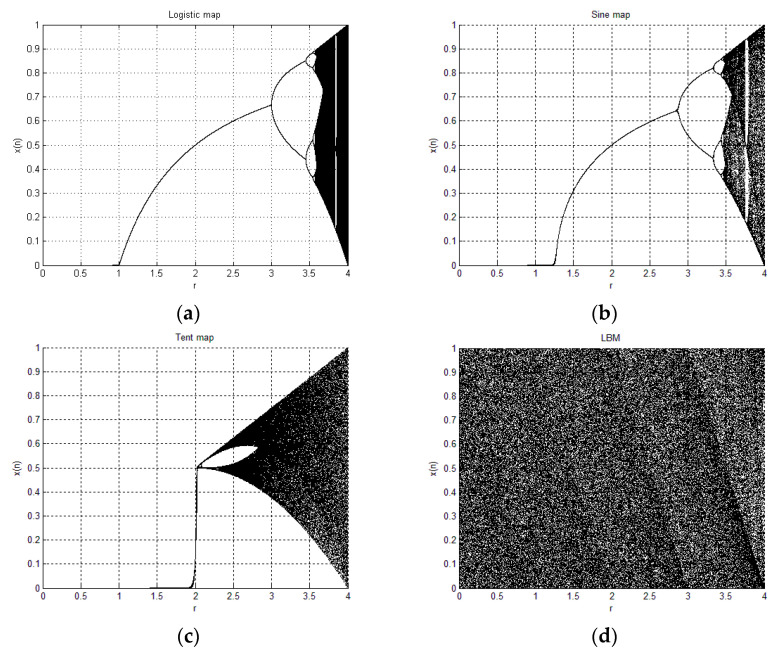
Bifurcation diagrams of (**a**) logistic map; (**b**) sine map; (**c**) tent map; (**d**) proposed logistic–Bernoulli map (LBM).

Like its bifurcation diagram shows (Figure 1), in most of the definitional domain of the coefficient, the logistic map has no chaotic behavior. When the coefficient *μ* is very close to 4, the dots can spread over the full range of *x_n_* in the bifurcation diagram, which indicates good chaotic properties. However, the proposed LBM, in the entire definitional domain [0, 4] of the coefficient *γ*, the system is chaotic, as shown in Figure 1d.

The bifurcation diagrams of the logistic map, sine map, tent map, and LBM are shown in Figure 1.

In [10], although the proposed 1D chaotic maps, including LTS, LSS, and TSS, can generate chaotic properties similar to that of LBM, the numbers of iterations required to calculate the random sequence with LTS, LSS, and TSS were higher than that of the proposed map. We iterate the proposed chaotic map, LTS, LSS, and TSS 10 times, and the average execution times are listed in Table 2. 

**Figure 2:**
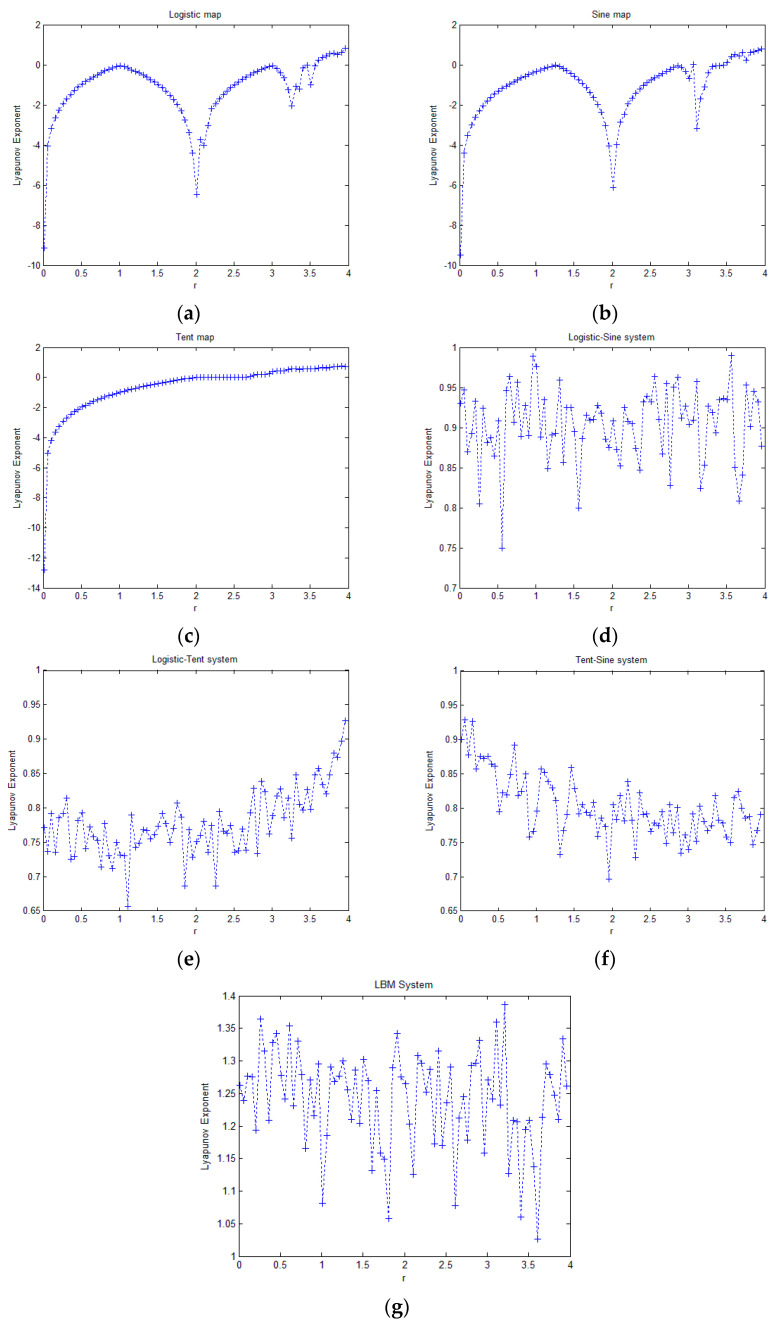
Lyapunov exponent of the (**a**) logistic map; (**b**) sine map; (**c**) tent map; (**d**) LSS; (**e**) LTS; (**f**) TSS; (**g**) proposed LBM.

To further investigate the chaotic properties of the proposed 1D chaotic map, we calculated and plotted the Lyapunov exponents of the proposed 1D chaotic map and comparison maps. A positive Lyapunov exponent indicates chaotic behavior, while a negative exponent indicates that there are no chaotic properties. A larger Lyapunov exponent indicates better chaotic properties. The maximum Lyapunov exponents for different parameters of the chaotic maps are plotted in Figure 2. 

As Figure 2 shows, the Lyapunov exponents of the logistic map, sine map, and tent map are larger than 0 when the parameter is very close to 4, and in most of domains of the parameter, there is no chaotic behavior at all. When the parameters of LSS, LTS, and TSS fall into [0, 4], the Lyapunov exponents are all larger than 0, indicating that over the entire parameter domain, these dynamical systems are chaotic. The Lyapunov exponents obtained by LBM were also larger than 0 over its entire parameter domain, and the Lyapunov exponents of LBM fall in the value range [1, 1.4] in all parameter domains. This range is higher than the range [0.7, 1] of LSS, LTS, TSS, which indicates that the proposed LBM system exhibits much better chaotic behavior.

To further test the randomness of the proposed LBM, the NIST (National Institute of Standards and Technology) SP800-22 test was performed on 300 random binary sequences generated by LBM, and the simulation results are shown in Table 3. This indicates that the random sequences have high randomness.

## 3. Cellular Automata-Based Diffusion Structure

The cellular automata algorithm provides a mechanism in which a slight change to the state of one cell in the current generation will lead to modification of all the cells’ states in the next generation. In an image-encryption algorithm, this feature makes cellular automata an ideal algorithm for the diffusion stage, as one main goal for diffusion is to spread a single slight modification of one pixel to all ciphered pixels. However, in previous cellular automata-based ciphers, cellular automata were employed only as a pseudorandom number generators, while neglecting the connection between diffusion effects and the intrinsic feature of cellular automata.

The idea of cellular automata was proposed by Stephen Wolfram in 1984 [27], and after that, a series of pseudorandom number generators using cellular automata were proposed [28]. A cellular automaton (CA) is a discrete model in the time and space domains, and each unit, which is called a cell, has a finite number of states. In the discrete time domain, each cell can evolve into any predefined state based on the neighboring cells’ states in a previous time slice, as defined in Equation (6) [28].

(6) $$a_{i}^{\prime }=\delta (a_{i-r},a_{i-r+1},\cdots ,a_{i+r}),$$

where i∈[0,M−1], *a_i_* is the current state between 0 and *k* − 1, and *r* is the number of neighbors in each direction that determine the current cell’s value. δ is the update rule of the CA. *a_i_*’ is the cell value in the next generation.

For a pseudorandom number-generation case, *k* = 2 and *r* = 1 are suggested in [28], and Equation (6) is transformed to Equation (7). The first number and the last number are calculated in a circular way, as shown in Equation (8) and Equation (9).

(7) $$a_{i}^{\prime }=\delta (a_{i-1},a_{i},a_{i+1}),$$

(8) $$a_{0}^{\prime }=\delta (a_{M-1},a_{0},a_{1}),$$

(9) $$a_{N-1}^{\prime }=\delta (a_{M-2},a_{M-1},a_{0}).$$

In this case, rules δ_90_ and δ_150_ are usually employed, as shown in Equations (10) and (11) [28].

(10) $$\mathrm{Rule}\;\delta_{90}:\;a_{i}^{t+1}=a_{i-1}^{t}⊕a_{i+1}^{t}$$

(11) $$\mathrm{Rule}\;\delta_{150}:\;a_{i}^{t+1}=a_{i-1}^{t}⊕a_{t}^{i}⊕a_{i+1}^{t}$$

When *k* = 2, the sequences generated by Equations (8) and (9) contain binary values. 

According to Equation (6), a CA with different generations can be defined by Equation (12).

(12) $$a_{i}^{t+1}=\delta (a_{{}_{i-r}}^{t},a_{{}_{i-r+1}}^{t},\cdots ,a_{i}^{t},\cdots ,a_{{}_{i+r}}^{t}),$$

where *t* is the generation number. Equation (12) illustrates that the state of cell *a_i_* in generation *t* + 1 is determined by three factors: the state of *a_i_* in generation *t*; the states of the *r* neighboring cells of *a_i_* in generation *t*; and the states of the *r* neighboring cells of *a_i_*. *r* is the radius of the influence range. 

As mentioned above, spreading the slight modification of one or several pixels to all the ciphered pixels is one significant characteristic for a diffusion algorithm. In a classical diffusion algorithm, this spreading mechanism is achieved in a linear way. The two-dimensional pixel array is firstly transformed into a one-dimensional array. The spreading starts at the modified pixel and ends at an unpredictable pixel, governed by the “stop-point mechanism”, which is discussed in our previous paper [29]; the end pixel is always not the last one. The finite number of influenced pixels can be treated as the seeds for the next round of diffusion, to influence more pixels in a backward manner (one direction).

**Figure 3:**
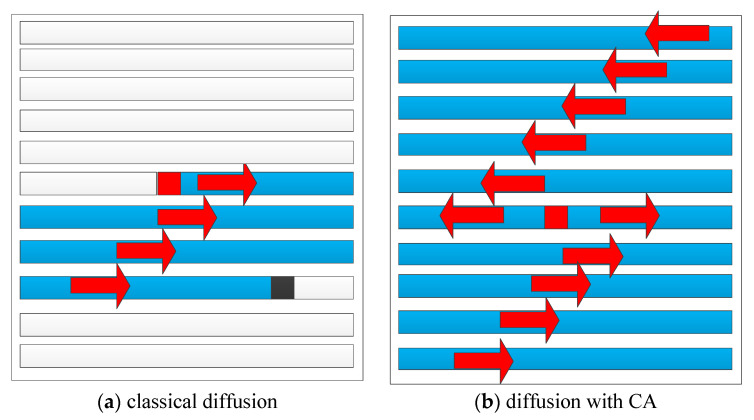
Mechanisms of classical diffusion and cellular automaton (CA)-based diffusion.

Between generations, a CA can diffuse one cell’s modification to neighboring pixels in two directions within its influence region of predefined size. In an ideal situation, a CA has the ability to influence all the cells’ states in one round of evolution or diffusion. The mechanisms of classical diffusion and CA-based diffusion are shown in Figure 3.

In Figure 3, the red pixel indicates the modified pixel and the spreading starts at this pixel. The black pixel is the last pixel that the spreading reaches when classical diffusion is performed, and all white pixels are unchanged. All blue pixels are modified or influenced by the spreading. Figure 3 shows that a diffusion algorithm with CA can spread the modification in two directions, and in each influence calculation, more pixels are affected according to Equation (12). Therefore, in the proposed scheme, we investigate if a new CA-based diffusion algorithm will lead to better spreading effects and make the system more sensitive.

## 4. The Proposed Scheme

In his masterpiece, Fridrich firstly proposed an image-encryption algorithm based on chaotic systems considering the similarities between cryptography and chaotic dynamic systems [9]. Two stages, confusion and diffusion, constitute one encryption round. Both stages are performed at the pixel level. Confusion operations aim to change each pixel’s location, while in diffusion stages, pixel values are modified. 

In [19], bit-level permutation is firstly proposed. In this permutation algorithm, the basic permuting unit is the bit, rather than the pixel, in the image. Each pixel contains eight bits of information. When the bit locations are changed, not only the pixel values but also the pixel locations are modified. When applying bit-level permutation, the plain image is firstly divided into eight-bit planes, and the eight-bit planes are permuted independently. After that, the eight permuted bit planes are joined together to generate the permuted cipher image. 

In most bit-level image ciphers, only confusion operations are performed on bit level, while in the diffusion phase, the calculations are performed on the pixel level [19,25,30]. Between the two phases, a transformation is required. In [20], the bit-level properties of digital images are investigated, and the following suggestions are provided. (1) The bit-level permutation algorithm should allow each bit to freely move to any position in any bit plane of any color channel. (2) From the perspective of the bit constitution of an image, the bit distributions (the numbers of 0 s or 1 s) of the plain image and cipher image do not significantly change. On average, only 3.3% of the bits of a plain image are modified during the encryption operations, and most of them only change locations. According to these suggestions, we design a new image-encryption algorithm using lightweight bit-level permutation and bit-level diffusion based on the cellular automata strategy to achieve a balance between efficiency and security.

### 4.1. Confusion of the Proposed Scheme

In the confusion stage, bit-level permutations are employed due to their superior features to relocate bit information of plain images. During the confusion operations, the LBM system defined by Equation (5) is used as the pseudorandom number generator.

**Figure 4:**
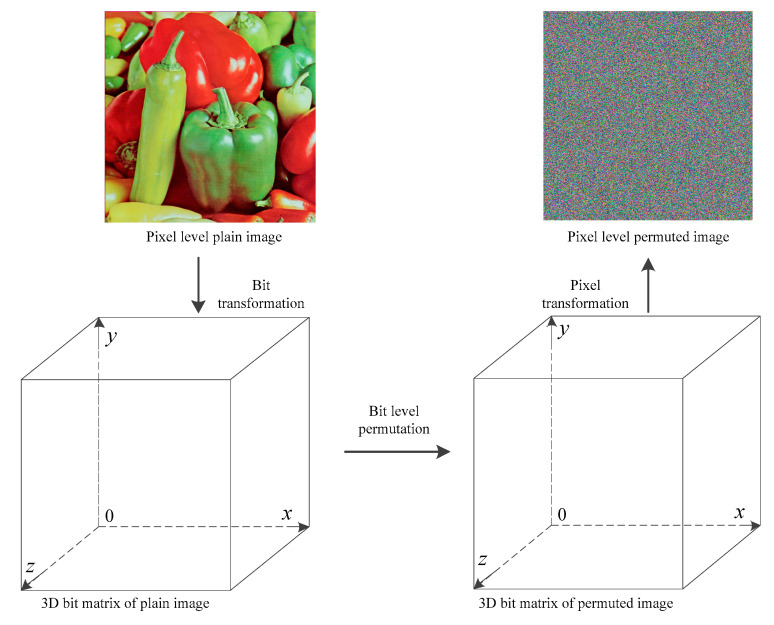
Bit-level permutation.

At the bit level, the image can be considered as a natural three-dimensional matrix, and the three dimensions are the width, height, and bit length, as shown in Figure 4.

As Figure 4 shows, in the first step, the pixel-level image is transformed into a three-dimensional (3D) matrix, on which the bit-level permutations are performed. The *x* and *y* coordinates of the 3D bit matrix correspond to the width and height of the plain image, respectively, while the *z* coordinate is the bit length of each pixel. Here, the width, height, and bit length are denoted by *w*_1_, *w*_2_, and *w*_3_.

In the second step, three random sequences are generated by LBM. When iterating the chaotic system, the generated random numbers are further quantified by Equation (13), and if the output random number is the same as a previously generated one, the number will be discarded.

(13) $$\left\{ \begin{array}{l} x_{i}^{\prime }=qu(x_{i})=(x_{i}\times 10^{5})\mathrm{mod}w_{1};\\ y_{j}^{\prime }=qu(y_{j})=(y_{j}\times 10^{5})\mathrm{mod}w_{2};\\ z_{k}^{\prime }=qu(y_{k})=(z_{k}\times 10^{5})\mathrm{mod}w_{3}. \end{array}\right.$$

In Equation (13), $i\in [0,w_{1})$, $j\in [0,w_{2})$, and $k\in [0,w_{3})$.

In the third step, the three random sequences obtained in Step 2 are sorted, and the three sorted sequences are denoted as *sx*’, *sy*’, and *sz*’, as shown in Equation (14).

(14) $$\left\{ \begin{array}{l} sx^{\prime }=sort(x^{\prime });\\ sy^{\prime }=sort(y^{\prime });\\ sz^{\prime }=sort(z^{\prime }). \end{array}\right.$$

By comparing *x*’ with its sorted sequence *sx*’, *y*’ with *sy*’, *z*’ with *sz*’, the three mapping rules can be obtained and applied on the three-dimensional bit matrix, as shown in Figure 7, and the permuted 3D bit matrix can be obtained.

### 4.2. Bit-Level Diffusion Using Cellular Automata

In our cryptosystem, Rule 90 is used due to its simple structure and high efficiency when updating the random sequence. When calculating the first and last numbers, Equations (8) and (9) are employed. 

In the first step of the diffusion stage, the permuted 3D bit matrix is divided into several diffusion bit planes for further diffusion operations. Let the size of the permuted 3D bit matrix be *m* × *n* × l, where *m* and *n* are the width and height of the image and *l* is the bit length of each pixel; *l* equals 24 when the plain image is an RGB color image. Since the diffusion bit planes have the same width and height as the plain image, the size of each can be defined as *m* × *n* × *sl*, where *sl* is the bit length of each unit (or pixel) in the diffusion bit plane. For example, if *sl* equals 1, there are 24 diffusion planes. The variable *pn* is the number of diffusion planes, which is defined by Equation (15).

(15) $$pn=\frac{24}{sl}$$

As Equation (15) shows, if each unit or pixel in a diffusion plane has 4 bits (*sl* = 4), the number of diffusion planes is 6. In the diffusion phase of the cryptosystem, *sl* can be randomly selected from among 1, 2, 4, 8, and 12. 

In the second step of the diffusion process, the proposed LBM defined in Equation (5) is used to generate two random sequences *f*_1_(*x*) and *f*_2_(*x*), where x∈[0,N×N−1]. The two sequences are further quantified by Equations (16) and (17).

(16) $$rdmf_{1}(i)=\left\lfloor \left(f_{1}(i)\times 100\right)\right\rfloor \mathrm{mod}2^{sl},$$

(17) $$rdm_{1}(i)=\left\lfloor \left(f_{2}(i)\times 100\right)\right\rfloor \mathrm{mod}2^{sl},$$

where i∈[0,N×N−1].

**Figure 5:**
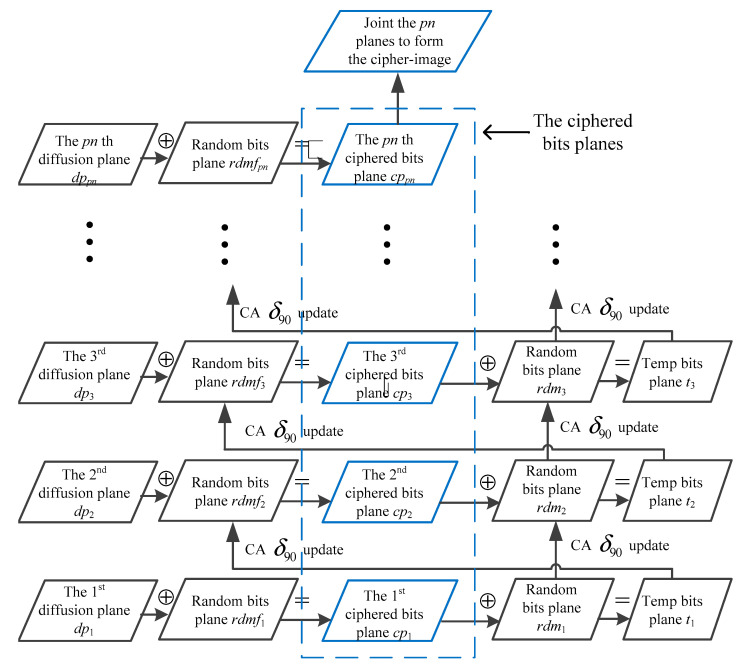
Diffusion procedure using cellular automata.

The diffusion operations are shown in Figure 5 and defined by Equation (18).

(18) $$\left\{ \begin{array}{l} cp_{s}(i)=dp_{s}(i)⊕rdmf_{s}(i);\\ t_{s}(i)=cp_{s}(i)⊕rdm_{s}(i);\\ rdmf_{s+1}=\delta_{90}(t_{s});\\ rdm_{s+1}=\delta_{90}(rdm_{s}). \end{array}\right.$$

In Equation (18), *dp_s_*(*i*) is the *i*th pixel in the *s*th diffusion plane of the permuted image, while *rdmf_s_*(*i*) is the *i*th random number in the *s*th random sequence, where s∈{1,2,…,pn}. *cp_s_*(*i*) denotes the *i*th pixel in the ciphered *s*th plane. *t_s_* is a temporary value sequence. In the 3rd and 4th equations of (18), we evolve the states of cells from $\left\{t_{s}(i)|i\in [0,N\times N-1]\right\}$ to $\left\{rdmf_{s+1}(i)|i\in [0,N\times N-1]\right\}$, and from $\left\{rdm_{s}(i)|i\in [0,N\times N-1]\right\}$ to $\left\{rdm_{s+1}(i)|i\in [0,N\times N-1]\right\}$, respectively, according to CA rule 90, as shown in Equation (10).

In the diffusion phase, different diffusion planes are treated as different generations for CA, and the bit-level diffusion structure is as shown in Figure 5. 

In Figure 5, if we consider each row of quadrangles (including two XOR (exclusive-OR) operations and two CA $\delta_{90}$ updating operations) as one step in the diffusion phase and let *sl* equal 1, there are a total of 24 diffusion planes and 24 steps in the diffusion phase. If *sl* equals 12, there are three steps in the diffusion phase. In Figure 5, the ciphered bit planes are marked by blue quadrangles. Taking the 2nd diffusion plane *dp*_2_ as an example, *dp*_2_ is firstly XORed with a random bit plane *rdmf*_2_, which is obtained by cellular automata with 90 update rules applied on *t*_1_, as shown in Figure 5. After that, the corresponding cipher diffusion plane *cp*_2_ is obtained. To calculate *t*_2_ for the next step of the diffusion phase, the obtained *cp*_2_ is further XORed with another random plane *rdm*_2_. *rdm*_2_ is calculated by cellular automata with 90 update rules applied on *rdm*_1_. With the help of *t_i_*, *rdm_i_*, *rdmf_i_*, and the $\delta_{90}$ updating rule, the current ciphered plane *cp_i_* has a strong relation with the previous encrypted cipher plane *cp_i_*_−1_, which makes the cryptosystem more sensitive and secure to the slight modification of the plain image during a differential attack.

## 5. Simulation Results and Security Analysis

The proposed image-encryption algorithm can be applied on both color images and grey-level images. When encrypting a grey-level image, it can be considered as one color channel of the color image. In this section, results of the simulations and security analysis are reported. All experiments are performed on a personal computer with Windows 7 Ultimate, Intel 3.4 GHz Dual-core CPU, and 8 GB memory, and the compiling environment is Visual C++ 2010. Some graphics are plotted in MATLAB 2009a. In this section, *sl* in Equation (15) is set to 8.

### 5.1. Simulation Results and Statistical Data Analysis

**Figure 6:**
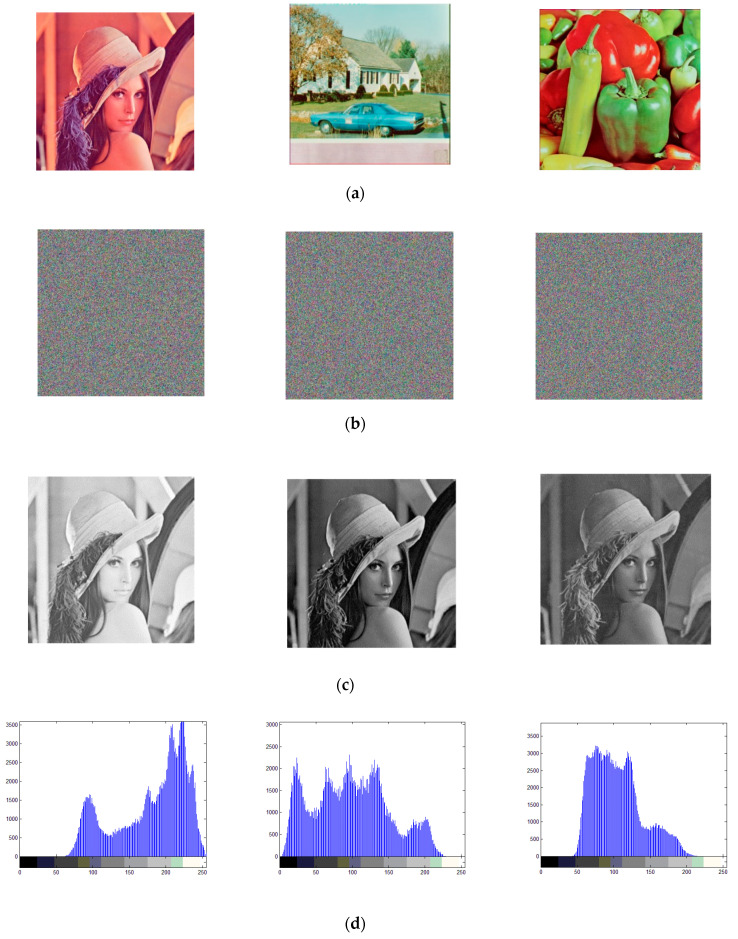

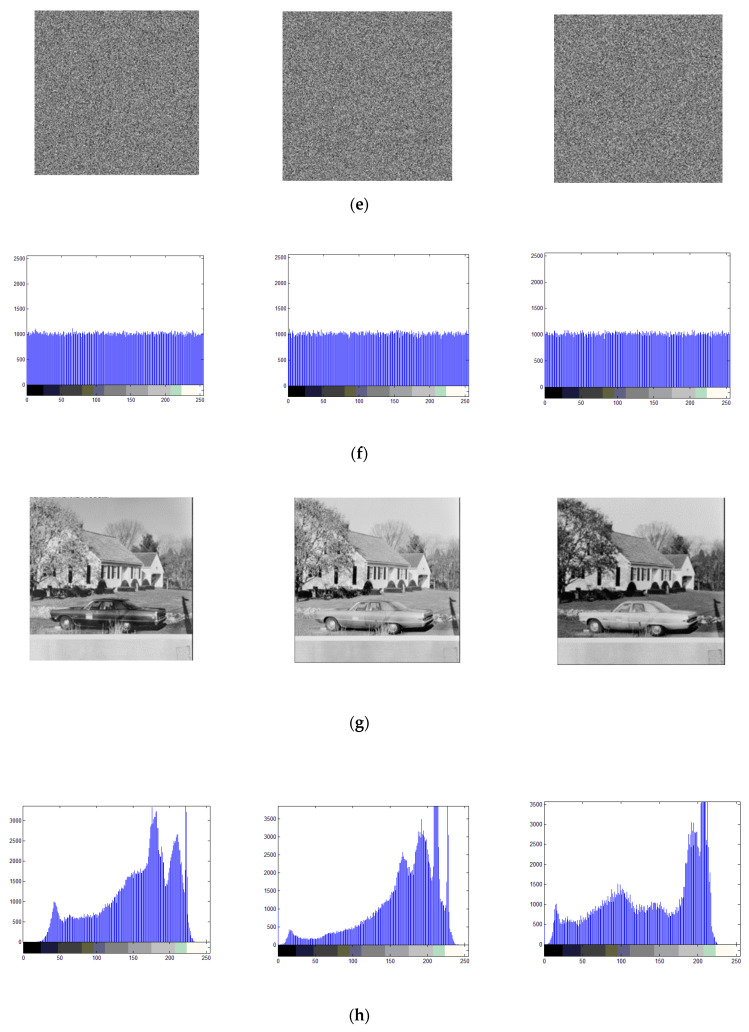

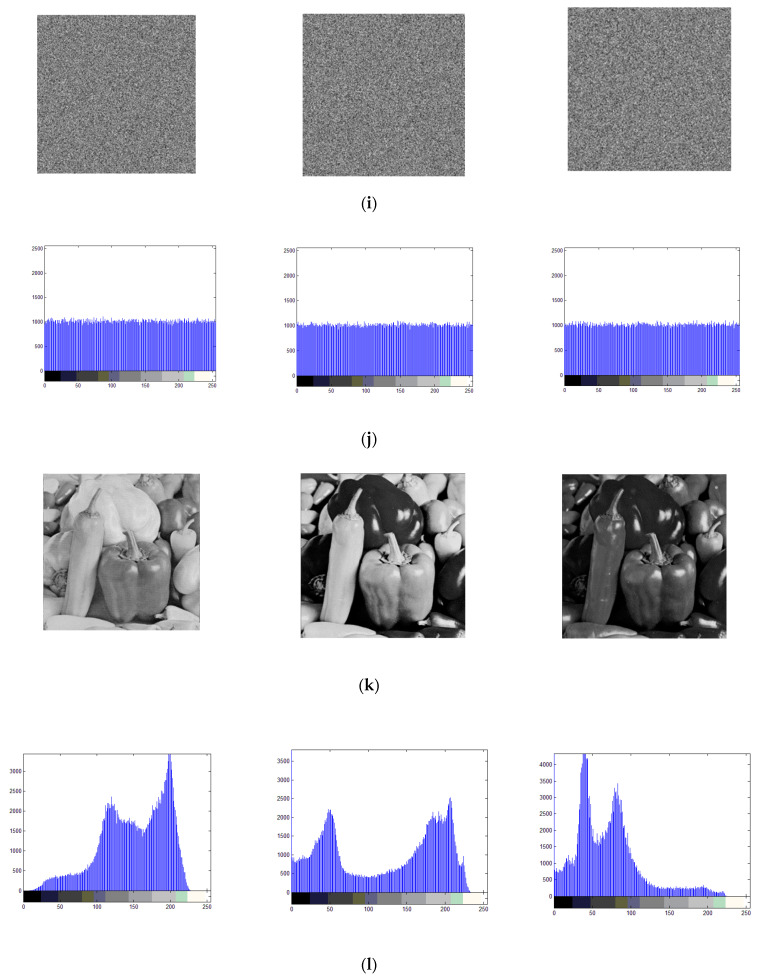

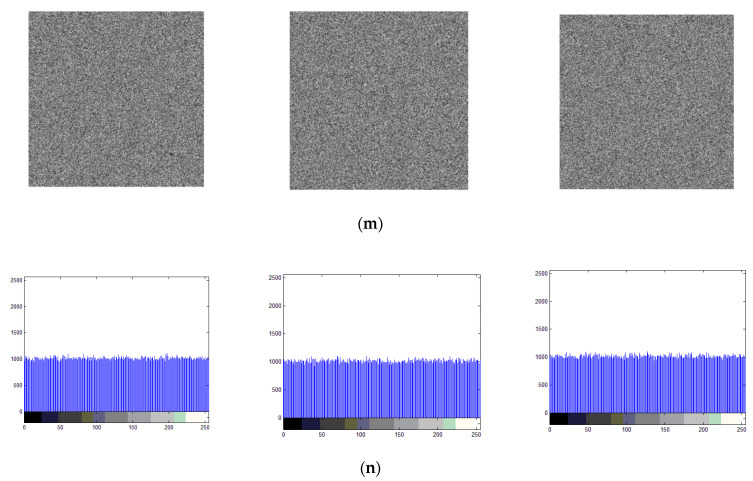
Simulation results and histogram analysis, figures in (**a**) are the plain images, figures in (**b**) are the cipher images, figures in (**c**) are the RGB channels of plain image Lena, figures in (**d**) are the histograms of plain image Lena, figures in (**e**) are the RGB channels of cipher image Lena, figures in (**f**) are the histograms of cipher image Lena, figures in (**g**) are the RGB channels of plain image house, figures in (**h**) are the histograms of plain image house, figures in (**i**) are the RGB channels of cipher image house, figures in (**j**) are the histograms of cipher image house, figures in (**k**) are the RGB channels of plain image peppers, figures in (**l**) are the histograms of plain image peppers, figures in (**m**) are the RGB channels of cipher image peppers, figures in (**n**) are the histograms of cipher image peppers.

The cipher images obtained by the proposed cryptosystem and their corresponding histograms are as shown in Figure 6.

The histogram of an image illustrates the pixel-value distribution information. For a meaningful image, the histogram always fluctuates and exhibits some gathering effects in some domains, while in a cipher image, all pixel values should be evenly distributed and completely different from those of the plain image. As Figure 6 shows, three test images, namely Lena, a house, and peppers, are encrypted by the proposed scheme, and their cipher images are shown in Figure 6b. We can see that the cipher image and the three corresponding color channels are all noisy, and the histograms of the three color channels in the cipher images are all in ideal situations, which indicates the high security of the proposed scheme through the statistical analysis.

In his masterpiece, Shannon noted that many ciphers can be cryptoanalyzed using statistical information, including histograms and correlation analysis [30]. For a good cipher, not only should the histogram of the cipher image be in the ideal situation, but the correlation coefficients of the cipher image should also be significantly reduced. Therefore, an important part of the statistical analysis is the correlation analysis.

The adjacent pixel values in the plain image are quite similar, and the correlation coefficients in any direction are all very close to 1. However, in the cipher image, there is no meaningful pattern and the correlation among adjacent pixels should be significantly reduced. In an ideal situation, the correlation coefficients should be close to 0. The correlation coefficients are calculated according to Equations (19)–(21). The correlation coefficients of the cipher image obtained by the proposed scheme are listed in Table 4.

(19) $$r_{x,y}=\frac{E\{[x-E(x)][y-E(y)]\}}{\sqrt{D(x)}\sqrt{D(y)}}$$

(20) $$E(x)=\frac{1}{S}\sum_{i=1}^{S}x_{i}$$

(21) $$D(x)=\frac{1}{S}\sum_{i=1}^{S}{\left[x_{i}-E(x)\right]}^{2}$$

In Table 4, the correlation coefficients in the horizontal, vertical, and diagonal directions of Lena, house, and peppers are very close to 1, indicating high correlations between the adjacent pixels in the plain image. However, the correlation coefficients of the cipher image are all close to 0; thus, the encryption operations have significantly reduced the correlation relationships. In Table 4, the correlation coefficients of the cipher image obtained by the proposed scheme are compared with those obtained by [10]; better values are marked in bold. The average absolute value of the correlation coefficients of the cipher image obtained by the proposed scheme is 0.002405, while the average absolute value for the comparison scheme is 0.005851. 

**Figure 7:**
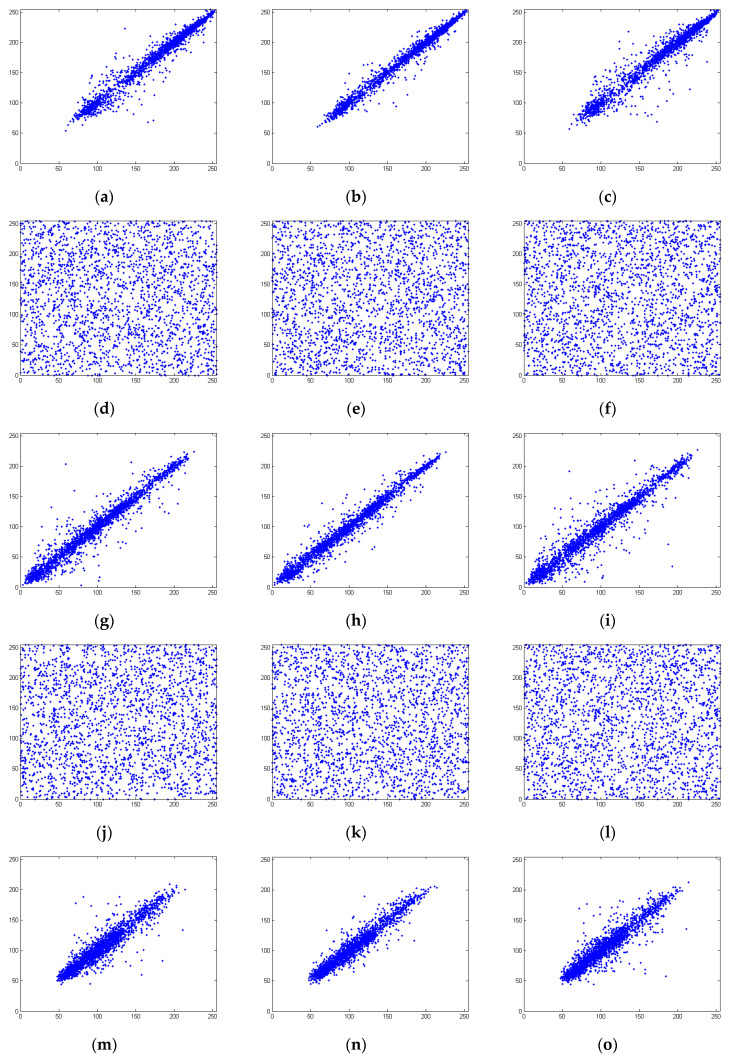

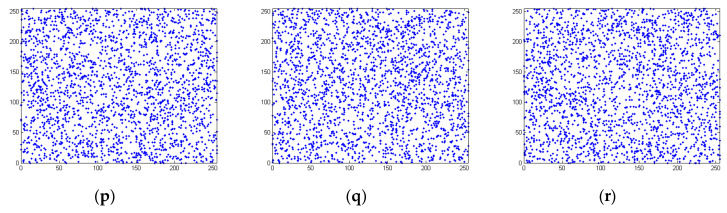
Correlation plots of the R channel of the plain image in the (**a**) horizontal, (**b**) vertical, and (**c**) diagonal directions; G channel of the plain image in the (**g**) horizontal, (**h**) vertical, and (**i**) diagonal directions; B channel of the plain image in the (**m**) horizontal, (**n**) vertical, and (**o**) diagonal directions; R channel of the cipher image in the (**d**) horizontal, (**e**) vertical, and (**f**) diagonal directions; G channel of the cipher image in the (**j**) horizontal, (**k**) vertical, and (**l**) diagonal directions; B channel of the cipher image in the (**p**) horizontal, (**q**) vertical, and (**r**) diagonal directions.

To graphically illustrate that the encryption operations significantly reduced the correlation relationships, the correlation distributions are plotted in Figure 7.

### 5.2. Differential-Attack Analysis

In a differential attack, the opponent will perform a series of encryption rounds with different plain images. In each round of the attack, the plain image will be slightly modified by one bit or one pixel, and after encryption, one pair consisting of the modified plain image and corresponding cipher image can be obtained. By analyzing several pairs of modified plain image with the corresponding cipher image, the equivalent key of the cryptosystem can be obtained. Therefore, to resist a differential attack, the cryptosystem should provide sufficient sensitivity to tiny modifications of the plain image. In other words, one slight modification to the plain image should lead to a completely different cipher image. Two measures are usually used in differential-attack analysis. They are NPCR (number of pixels change rate) and UACI (unified average changing intensity). NPCR and UACI are defined by Equations (22) and (23). In the simulations, the last pixel of the G channel of the plain image is modified. The NPCR and UACI data are listed in Table 5 and Table 6.

(22) $$NPCR=\frac{\sum_{i,j}D(i,j)}{M\times N}\times 100\%,$$

where *D*(*i*, *j*) is defined as

$$D(i,j)=\left\{ \begin{array}{l} 1,\;c_{1}(i,j)\neq c_{2}(i,j)\\ 0,\;otherwise \end{array}\right.,$$

and *c*_1_ and *c*_2_ are the two cipher images.

(23) $$UACI=\frac{1}{M\times N}\left[\sum_{i,j}\frac{\left|c_{1}(i,j)-c_{2}(i,j)\right|}{255}\right]\times 100\%$$

### 5.3. Information-Entropy Analysis

Information entropy is a significant criterion for measuring the security strength of a symmetric cryptosystem. The definition of the information entropy *H*(*m*) of a source m is given in Equation (24).

(24) $$H(m)=\sum_{i=0}^{2^{N}-1}p(m_{i})\log\frac{1}{p(m_{i})}$$

In Equation (24), *p*(*m_i_*) is the probability of symbol *m_i_* and log denotes the base 2 logarithm. If each symbol in the message source *m* is an eight-bit symbol and the 256 possible outcomes each have equal probability, the message can be considered random information, and in this case, the information entropy should be 8, which is the ideal situation of a cipher text obtained by a cryptosystem.

The information entropies of the proposed scheme and a comparable scheme are reported in Table 7.

Table 7 shows that the information entropies of the cipher image obtained by the proposed scheme are larger than those of the comparison scheme, and are very close to the ideal value of 8. This indicates that the cipher image does not leak any of the information in the original plain image.

### 5.4. Speed Test

Computational efficiency is another significant issue for a cryptosystem. To evaluate the encryption speed of the proposed scheme, the test image “Lena” is encrypted 10 times, and the average encryption times are listed in Table 8.

As there are no apparent confusion and diffusion stages in [10], only the total time of the encryption algorithm in [10] is provided in Table 8. The results show that our scheme is faster than the comparable one.

## 6. Conclusions

A new one-dimensional chaotic logistic–Bernoulli map is proposed and investigated. The new map shows superior features in chaotic behavior and efficiency. In addition, a new diffusion structure using a cellular automata strategy is proposed. Based on the new chaotic map and diffusion structure, a new image-encryption algorithm is proposed. Simulation results show that the proposed scheme leads to a higher level of security and is computationally efficient.

## Figures and Tables

**Table 1:** The speed comparison among the logistic map, sine map, tent map, and Bernoulli map.

	Logistic Map	Sine Map	Tent Map	Bernoulli Map
Generating 3,000,000 random numbers	30.9 ms	96.2 ms	48.8 ms	48.0 ms

**Table 2:** Speed comparison among logistic–tent system (LTS), logistic–sine system (LSS), tent–sine system (TSS), and the proposed LBM.

	LTS [10]	LSS [10]	TSS [10]	LBM (Proposed)
Generating 3,000,000 random numbers	81.5 ms	128.2 ms	143.9 ms	53.0 ms

**Table 3:** Randomness test results of 300 random sequences generated by LBM using NIST.

Statistical Tests	*P*-Value ≧ 0.01		Pass Rate (%) ≧ 0.9628	
Frequency	0.280306	Pass	98.67	Pass
Block frequency	0.685579	Pass	99.00	Pass
Cumulative sums	0.748094	Pass	98.83	Pass
Runs	0.032923	Pass	99.33	Pass
Longest run	0.534146	Pass	98.33	Pass
Rank	0.425059	Pass	99.67	Pass
FFT	0.804337	Pass	98.33	Pass
Non overlapping template	0.485302	Pass	98.96	Pass
Overlapping template	0.294431	Pass	99.67	Pass
Universal	0.057753	Pass	100.00	Pass
Approximate entropy	0.739918	Pass	99.67	Pass
Random excursions	0.648693	Pass	98.73	Pass
Random excursions variant	0.588852	Pass	98.81	Pass
Serial	0.515315	Pass	99.33	Pass
Linear complexity	0.561227	Pass	99.33	Pass

**Table 4:** Correlation coefficients of the proposed scheme.

Test Image	Color Channel	Direction	Plain Image	Cipher Image	Cipher Image Obtained by [10]
Lena	R channel	Horizontal	0.980042	−0.006439	0.009238
		Vertical	0.989399	−0.001656	0.001778
		Diagonal	0.969445	0.007549	0.004084
	G channel	Horizontal	0.968884	−0.002014	0.0062322
		Vertical	0.982293	0.000549	0.0076567
		Diagonal	0.955277	0.001466	0.0053490
	B channel	Horizontal	0.933162	0.000000	0.0073123
		Vertical	0.958172	−0.001465	0.0067766
		Diagonal	0.918504	−0.001098	−0.0051781
House	R channel	Horizontal	0.954274	−0.000549	0.004812
		Vertical	0.953098	0.000916	0.000535
		Diagonal	0.918540	−0.004959	0.001604
	G channel	Horizontal	0.933637	−0.006428	0.012478
		Vertical	0.927962	0.001653	0.006774
		Diagonal	0.871893	0.000917	0.006414
	B channel	Horizontal	0.975040	−0.002933	0.011060
		Vertical	0.959132	−0.002566	0.007135
		Diagonal	0.938001	−0.001280	−0.001784
Peppers	R channel	Horizontal	0.958355	0.003853	0.005328
		Vertical	0.964731	0.001284	0.004085
		Diagonal	0.949474	−0.001832	0.006211
	G channel	Horizontal	0.977986	−0.000912	0.008552
		Vertical	0.977363	0.001460	0.009086
		Diagonal	0.961298	0.002366	0.000178
	B channel	Horizontal	0.964817	−0.001647	0.008550
		Vertical	0.960795	0.006770	0.009263
		Diagonal	0.940662	−0.000366	−0.000534
Average absolute value				0.002405	0.005851

**Table 5:** The number of pixels change rate (NPCR) performances of proposed scheme and a comparable scheme.

Encryption Round	Proposed Scheme	NPCR of [10]
1	97.4969	0.0002543
2	99.6085	0.0005086
3	99.5963	0.0005086
4	99.6042	0.0005086
5	99.6033	0.0010173

**Table 6:** The unified average changing intensity (UACI) performances of proposed scheme and a comparable scheme.

Encryption Round	Proposed Scheme	UACI of [10]
1	32.6231	0.0001865
2	33.4585	0.0002314
3	33.4285	0.0002164
4	33.4867	0.0001157
5	33.4933	0.0005256

**Table 7:** Information entropies of the proposed scheme and a comparable scheme.

Encryption Round	Color Channel	Entropy (Lena)		Entropy (House)		Entropy (Peppers)	
		Proposed	[10]	Proposed	[10]	Proposed	[10]
1	R	7.999323	7.996066	7.999322	7.996852	7.999212	7.996819
	G	7.999260	7.996701	7.999304	7.996157	7.999331	7.996416
	B	7.999418	7.997363	7.999249	7.996136	7.999314	7.996867

**Table 8:** Speed performance.

Encryption Algorithm	Average Confusion Time (ms)	Average Diffusion Time (ms)	Average Total Time (ms)
Proposed scheme	82	30	112
The scheme in [10]	-	-	200 ms
